# Clinical Course of Severe Perineal Hypospadias with Cryptorchid Testicular Tumors in a Dog: Contextual Reference to Developmental and Endocrine Transcriptomic Pathways

**DOI:** 10.3390/cimb48050455

**Published:** 2026-04-28

**Authors:** Nuri Lee, Kibum Kwon, Ahsa Oh, Kyuhyung Choi

**Affiliations:** 1 Yeeun Animal Hospital, Seoul 06052, Republic of Korea; inuri1991@yonsei.ac.kr (N.L.); yeah4u@naver.com (K.K.); iamahssa@gmail.com (A.O.); 2 Department of Medical Device Engineering and Management, Yonsei University College of Medicine, Seoul 03722, Republic of Korea; 3 Bundang New York Animal Hospital, Seongnam 13637, Republic of Korea; 4 Department of Medical Informatics, College of Medicine, The Catholic University of Korea, Seoul 06591, Republic of Korea

**Keywords:** hypospadias, cryptorchid testicular neoplasia, developmental urogenital biology, endocrine tumorigenesis, translational transcriptomic context

## Abstract

Hypospadias is a congenital malformation of the male external genitalia resulting from incomplete fusion of the urethral folds during embryonic development. The perineal form represents the most severe phenotype and is frequently associated with abnormalities such as cryptorchidism and penile hypoplasia. Although surgical correction is generally recommended in young dogs, the long-term clinical course of severe hypospadias under conservative management remains poorly documented. In this study, we describe an unusual canine case of severe perineal hypospadias that survived to geriatric age under conservative management and subsequently developed bilateral testicular tumors arising from cryptorchid testes. Despite recurrent urinary tract infections during early life, the patient maintained an acceptable quality of life with long-term supportive care, providing a rare clinical example of extended survival without surgical correction. Because no molecular material was available from the patient, publicly available mouse transcriptomic datasets related to genital tubercle development and Leydig cell differentiation were consulted only as contextual reference. These datasets illustrate established developmental regulators and steroidogenic pathways relevant to genital formation and testicular function but do not represent direct molecular findings from the reported case. This report primarily highlights the clinical course and management of severe hypospadias in a dog, while using existing transcriptomic knowledge solely to provide biological context. The findings should therefore be interpreted as descriptive and hypothesis-generating rather than as evidence of a direct mechanistic link between developmental abnormalities and endocrine tumorigenesis.

## 1. Introduction

Hypospadias is a congenital malformation of the male external genitalia characterized by an ectopic urethral opening along the ventral surface of the penis, scrotum, or perineum [1]. The condition arises from incomplete fusion of the urethral folds during embryonic development and is classified according to the position of the urethral meatus [2]. Among these forms, perineal hypospadias represents the most severe phenotype, often accompanied by additional abnormalities including penile hypoplasia, cryptorchidism, and malformations of the prepuce [3].

Although hypospadias has traditionally been considered uncommon in dogs [4], its true prevalence may be underestimated [5] because detailed examination of the external genitalia is not always performed during routine veterinary examinations. Clinical consequences may include recurrent urinary tract infections, urine scald dermatitis, and chronic irritation of exposed mucosa [6]. For these reasons, surgical correction is generally recommended during early life [7]. However, conservative management may be considered in selected cases when severe clinical complications are absent. Despite this possibility, long-term outcomes of animals with severe hypospadias managed without surgery remain poorly documented [8].

The embryonic development of the external genitalia is tightly regulated by androgen signaling and developmental transcriptional programs. Formation of the genital tubercle involves coordinated activity of several morphogenetic pathways, including HOX transcription factors, sonic hedgehog (SHH) signaling [9], WNT signaling [10], and fibroblast growth factor pathways [11]. These pathways regulate epithelial–mesenchymal interactions and tissue patterning during genital morphogenesis [12]. Disturbances in these developmental processes have been implicated in congenital urogenital anomalies such as hypospadias [13].

In addition to developmental patterning, androgen production by Leydig cells plays a critical role in male genital development. Steroidogenic enzymes including CYP11A1 and HSD3B1 catalyze essential steps in androgen biosynthesis, while the peptide hormone insulin-like peptide 3 (INSL3) [14,15,16] produced by Leydig cells is required for normal testicular descent. Disruption of these endocrine pathways may therefore contribute to abnormalities such as cryptorchidism and impaired reproductive development [17].

Cryptorchidism is of particular clinical importance because retained testes have a substantially increased risk of neoplastic transformation. Testicular tumors such as seminomas and Leydig cell tumors occur more frequently in cryptorchid testes than in normally descended testes. These tumors originate from germ cells and steroidogenic interstitial cells, respectively, and their development is closely associated with hormonal signaling pathways.

Despite these observations, the molecular relationship between congenital genital malformations, endocrine dysfunction, and testicular tumor development remains incompletely understood. In particular, it remains unclear whether disruption of androgen-dependent developmental pathways may link these phenomena [18].

In the present study, we describe an unusual canine case of severe perineal hypospadias that survived to geriatric age under conservative management and subsequently developed bilateral testicular tumors arising from cryptorchid testes. Because no molecular material was available from the patient, publicly available transcriptomic datasets were consulted only as contextual reference to illustrate established developmental and endocrine pathways relevant to genital development and Leydig cell function. Accordingly, the primary contribution of this study is clinical, whereas the transcriptomic component is intended solely as supportive biological background rather than as a source of novel molecular findings or species-specific conclusions.

## 2. Materials and Methods

### 2.1. Public Transcriptomic Datasets

Publicly available transcriptomic datasets were obtained from the Gene Expression Omnibus (GEO) database. Single-cell RNA sequencing data of embryonic genital tubercle tissue were obtained from accession GSE175498 (https://www.ncbi.nlm.nih.gov/geo/query/acc.cgi?acc=GSE175498, accessed on 11 March 2026). RNA sequencing data from a Leydig cell differentiation model were obtained from GSE275902 (https://www.ncbi.nlm.nih.gov/geo/query/acc.cgi?acc=GSE275902, accessed on 11 March 2026). All analyses were conducted locally using the R statistical environment (version 4.4.2). The transcriptomic analyses in this study were designed as a targeted, hypothesis-driven exploration focusing on predefined developmental and steroidogenic genes. These analyses were not intended as a comprehensive or discovery-driven bioinformatic investigation, but rather as a contextual framework to aid biological interpretation of the clinical case. These transcriptomic datasets were used as a biological reference framework to illustrate known developmental and endocrine pathways, rather than to derive novel molecular insights or species-specific conclusions.

### 2.2. Data Retrieval and Preprocessing

GEO datasets were downloaded programmatically using the GEOquery package in R version 4.4.2 (R Foundation for Statistical Computing, Vienna, Austria). Expression matrices, phenotype information, and feature annotations were extracted from ExpressionSet objects and saved as R data objects for downstream analyses.

For datasets containing multiple ExpressionSet entries, the dataset with the largest sample size and feature count was selected for analysis. Expression matrices were stored as gene-by-sample matrices, and associated metadata tables were extracted from GEO phenotype information.

Supplementary Files containing raw count matrices were downloaded when available. These files included Matrix Market formatted count matrices as well as barcode and feature annotation tables.

### 2.3. Processing of Single-Cell RNA-Seq Data

Raw count matrices for male and female genital tubercle samples were downloaded from the Supplementary Files of GSE175498. The raw matrices were imported using the Matrix::readMM function. Barcode and gene annotation tables were processed using the readr (version 2.1.5) and dplyr packages (version 1.1.4).

Separate matrices corresponding to male and female samples were merged into a single gene-by-cell count matrix. Gene identifiers were standardized using unique gene symbols to avoid duplicated feature names.

Cell-level metadata were generated from barcode information and sample annotations. Quality-control metrics including total counts per cell (nCount_RNA) and number of detected genes (nFeature_RNA) were calculated using sparse matrix operations.

### 2.4. Quality Control Filtering

Cells with extremely low transcript complexity were removed using minimal filtering thresholds to retain biologically meaningful cells.

The following criteria were applied:

nFeature_RNA ≥ 200

nCount_RNA ≥ 500

After filtering, 43,827 cells were retained for downstream analysis.

Gene-level metadata including total read counts and number of expressing cells were also calculated.

### 2.5. Single-Cell RNA-Seq Analysis

Single-cell analyses were performed using the Seurat package.

Gene expression values were normalized using the LogNormalize method with a scale factor of 10,000. Highly variable genes were identified using the vst method with 3000 features.

Data scaling was performed using the ScaleData function. Dimensionality reduction was conducted using principal component analysis (PCA).

A shared nearest-neighbor graph was constructed using the first 20 principal components. Cell clusters were identified using the FindClusters function with a resolution parameter of 0.4.

Two-dimensional visualization of the cellular transcriptional landscape was performed using Uniform Manifold Approximation and Projection (UMAP) [19].

Developmental and androgen-related marker genes were visualized using FeaturePlot and DotPlot functions. The single-cell RNA-seq analysis was limited to visualization of known developmental regulators and was not intended for exhaustive cell-type annotation or differential expression analysis.

### 2.6. Leydig Cell RNA-Seq Analysis

For the Leydig differentiation dataset (GSE275902), raw gene-level read counts were downloaded from GEO Supplementary Files. Gene expression counts were converted to counts per million (CPM) by normalizing to library size. Expression values were then transformed using log_2_(CPM + 1). Genes associated with androgen signaling and steroidogenesis were selected based on a predefined gene panel including:

AR

SRD5A2

NR5A1

STAR

CYP11A1

HSD3B1

INSL3

NR2F2

Principal component analysis was performed using the prcomp function to evaluate global transcriptional differences between experimental conditions. This analysis was restricted to a predefined set of biologically relevant genes and does not represent a global transcriptomic analysis.

### 2.7. Statistical Analysis

Expression differences between control and knockout samples were evaluated using Welch’s two-sample *t*-test, which does not assume equal variance between groups. To control for multiple hypothesis testing, Benjamini–Hochberg false discovery rate (FDR) correction was applied. Genes with FDR < 0.05 were considered statistically significant.

### 2.8. Data Visualization

Data visualization was performed using ggplot2, patchwork, and pheatmap packages.

Heatmaps were generated to visualize expression patterns of steroidogenic and androgen-related genes across samples.

Boxplots were used to illustrate expression differences between control and knockout groups, with individual sample values displayed as jittered points. Statistical significance values (FDR) were annotated directly on the plots.

Dimensionality reduction results from single-cell analyses were visualized using UMAP plots, and marker gene expression patterns were displayed using FeaturePlot and DotPlot functions.

### 2.9. Software Environment

All computational analyses were conducted using R version 4.4.2 on a local workstation. The primary R packages used in this study included:

GEOquery

Seurat

Matrix

dplyr

readr

ggplot2

patchwork

pheatmap

## 3. Results

### 3.1. Case Description and Long-Term Conservative Management of Severe Perineal Hypospadias

**Figure 1:**
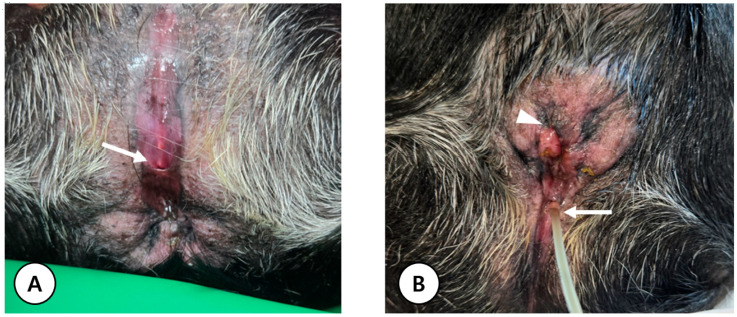
Perineal urethral opening associated with severe hypospadias. (**A**) Ventral view of the perineal region showing the ectopic urethral opening located ventral to the anus (white arrow). The penile mucosa is exposed because of incomplete preputial closure. (**B**) Dorsoventral view demonstrating the abnormal urethral meatus in the perineal region. A feeding tube inserted through the ectopic urethral opening (white arrow) confirms patency of the abnormal urethral tract. The arrowhead indicates inflamed perineal tissue surrounding the abnormal urethral opening.

**Figure 2:**
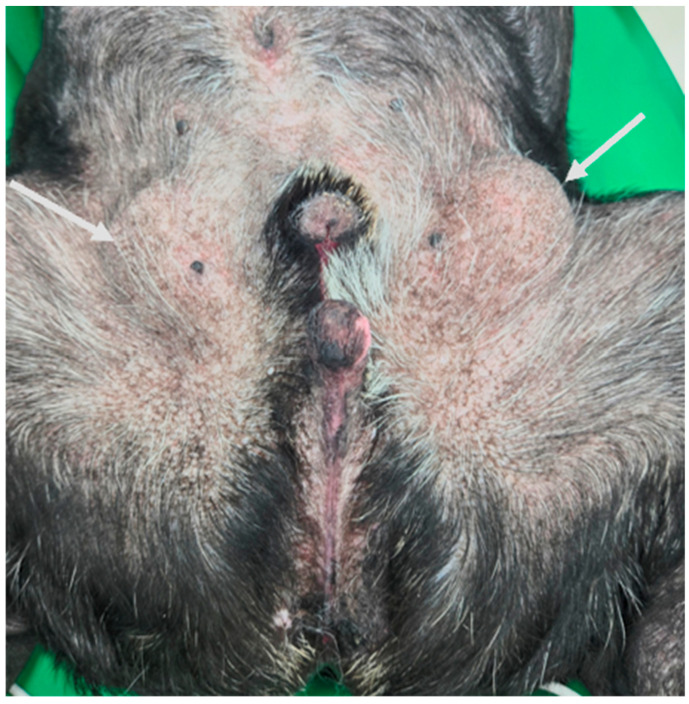
Physical examination findings. Ventral view of the caudal abdomen showing bilateral inguinal cryptorchid testes (white arrows). The retained testes are visible as symmetric inguinal swellings adjacent to the perineal region.

**Figure 3:**
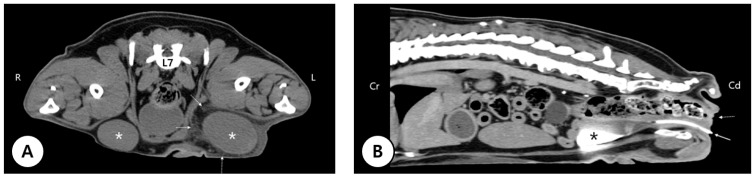
Computed tomography (CT) findings. (R = Right, L = Left, L7 = Seventh lumbar vertebrae, Cr = Cranial, Cd = Caudal) (**A**) L7 showing bilateral inguinal cryptorchid testes (asterisks). Increased surrounding soft tissue attenuation adjacent to the left testis (dashed arrow) is consistent with panniculitis. (**B**) Post-contrast sagittal CT image of the caudal abdomen demonstrating a shortened os penis and ectopic urethral opening in the perineal region (solid arrow). A urinary catheter inserted through the abnormal urethral orifice confirms patency of the ectopic tract. The asterisk indicates contrast medium within the urinary bladder. Dashed arrow indicates the anus.

**Figure 4:**
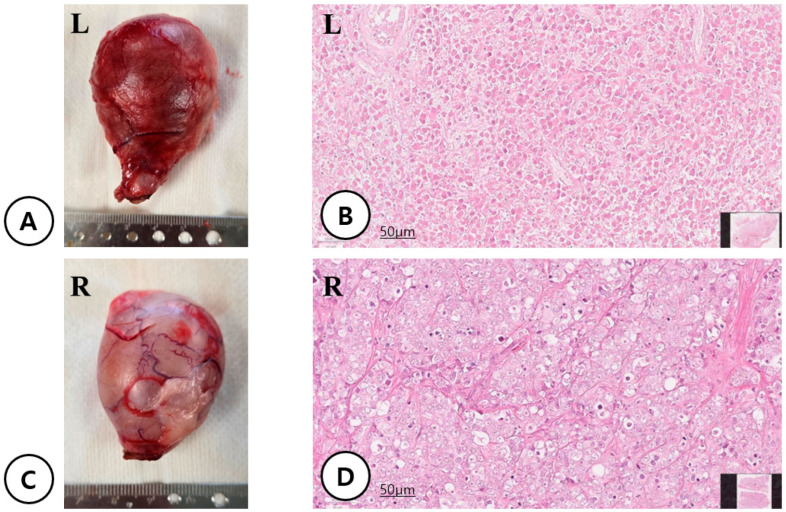
Gross and histopathological findings of bilateral cryptorchid testes. (L = Left, R = Right) (**A**) Gross appearance of the enlarged left cryptorchid testis forming a well-demarcated expansile mass lesion. (**B**) Histologically, the left testis is diffusely effaced by an unencapsulated but well-demarcated neoplasm composed of sheets and short interlacing cords of polygonal interstitial cells supported by delicate fibrovascular stroma. The neoplastic cells exhibit abundant finely granular eosinophilic cytoplasm and distinct cell borders. Nuclei are round to oval with finely stippled chromatin and inconspicuous to small nucleoli, showing mild to moderate anisokaryosis. Mitotic figures are rare (<1 per high-power field). Multifocal hemorrhagic to microcystic spaces are occasionally present. These findings are consistent with a Leydig cell tumor (H&E, ×200). (**C**) Gross appearance of the right cryptorchid testis following surgical excision. (**D**) Histologically, the right testis is replaced by a densely cellular, unencapsulated neoplasm arranged in sheets and lobules separated by thin fibrous septa. The neoplastic cells are large and round with moderate to abundant clear to lightly eosinophilic cytoplasm. Nuclei are centrally located with vesicular chromatin and prominent nucleoli. Marked mitotic activity is observed (frequently >5 per high-power field). Scattered small lymphocytes are interspersed within the tumor. Importantly, multifocal vascular invasion with intravascular tumor emboli is identified, supporting malignant potential. These features are consistent with seminoma (H&E, ×200).

An 11-year-old intact male French Bulldog (10.4 kg) was presented to Yeeun Animal Hospital (Seoul, Republic of Korea) with a rapidly enlarging inguinal cryptorchid testis associated with discomfort. Physical examination revealed incomplete closure of the prepuce, resulting in exposure of the penile mucosa. The penis was hypoplastic, and the urethral orifice was located in the perineal region ventral to the anus (Figure 1). The preputial covering was absent from the glans to the scrotal region, and the scrotum itself was absent. Both testes were retained within the inguinal region and were palpable on examination. Inflammatory changes consistent with panniculitis were noted surrounding the enlarged left testis (Figure 2). Computed tomography (CT) confirmed bilateral inguinal cryptorchid testes measuring 44.6 × 33.9 × 24.1 mm on the right and 50.4 × 36.5 × 31.6 mm on the left, with surrounding panniculitis adjacent to the left testis. Imaging also revealed a shortened os penis and an ectopic urethral opening in the perineal region (Figure 3). Contrast medium introduced through the perineal urethral orifice reached the urinary bladder via the ectopic meatus. However, urography and catheterization indicated no continuity between this tract and the normal prostatic urethra. No additional abnormalities or evidence of metastatic lesions were identified. Because of progressive enlargement of the retained testes, bilateral orchiectomy was performed. Histopathological examination revealed a Leydig cell tumor in the left testis and a seminoma in the right testis (Figure 4). Surgical correction of the hypospadias was not pursued because the dog had reached an advanced age and had adapted well to the congenital anomaly. The postoperative course was uneventful.

According to the owner, the dog had been diagnosed with perineal hypospadias shortly after birth. Survival during the neonatal period required meticulous management, including restriction of maternal licking and careful prevention of contamination of the ectopic urethral opening. During early life the dog experienced recurrent urinary tract infections (UTIs), which occurred intermittently until approximately six years of age and typically resolved with short courses of antimicrobial therapy. A prolonged infection occurred following swimming in a river, but thereafter the frequency of recurrence declined markedly, and no UTIs were reported during the five years preceding presentation.

With advancing age, the urinary stream gradually weakened, leading to occasional urine contamination of the hind limbs and necessitating more intensive hygienic care. Behaviorally, the dog displayed pronounced urine-marking behavior during walks and occasionally exhibited anxiety or aggression when other dogs attempted to sniff the genital region.

Long-term management consisted primarily of daily cleansing of the perineal region and urethral opening. During suspected UTI episodes or minor perineal injuries, chlorhexidine irrigation combined with antimicrobial therapy was used. After seven years of age, the dog was transitioned to a senior-care dietary regimen consisting of home-prepared meals enriched with vegetables and supplemented with probiotics and omega-3 fatty acids. Under this long-term conservative management strategy, the patient remained clinically stable and maintained a good quality of life until presentation at 11 years of age, despite the presence of severe congenital urogenital malformation.

### 3.2. Contextual Reference to Developmental Pathways in Public Mouse Genital Tubercle Transcriptomic Data

To investigate transcriptional programs associated with urogenital development, single-cell RNA sequencing data from embryonic genital tubercle tissue (GSE175498) were analyzed. After quality-control filtering based on transcript complexity thresholds (nFeature ≥ 200 and nCount ≥ 500), a total of 43,827 cells were retained for downstream analysis.

Dimensionality reduction using principal component analysis followed by Uniform Manifold Approximation and Projection (UMAP) revealed 16 transcriptionally distinct cell clusters, representing heterogeneous cellular populations within the developing genital tubercle (Supplementary Figure S1A). These clusters likely correspond to multiple developmental lineages involved in morphogenesis of the external genitalia.

When cells were colored according to biological sex, male and female cells were largely intermingled across the UMAP space. This pattern suggests that early transcriptional programs of genital tubercle development are largely shared between sexes at this stage (Supplementary Figure S1B).

To characterize developmental signaling pathways within the tissue, expression patterns of key developmental regulators were examined using FeaturePlot visualization. Several genes known to play critical roles in genital tubercle morphogenesis were detected across distinct cellular populations. In particular, HOXA13 and HOXD13, members of the HOX transcription factor family, exhibited widespread expression across multiple clusters. These genes are well-established regulators of distal limb and genital development. Additional morphogenetic signaling molecules were also detected in specific cell populations. Expression of SHH, WNT5A, FGF8, and BMP7 highlighted transcriptional programs associated with epithelial–mesenchymal signaling and pattern formation during genital tubercle development. These pathways are known to coordinate cell proliferation, differentiation, and tissue patterning during formation of the external genitalia (Supplementary Figure S1C–J).

In addition to developmental regulators, genes related to androgen signaling were also detected. Expression of the androgen receptor gene AR and the steroid metabolism gene SRD5A2 was observed in several clusters, suggesting localized androgen responsiveness within the developing tissue.

Dot-plot visualization summarized the distribution of developmental and androgen-related genes across clusters. Developmental patterning genes such as HOXA13, HOXD13, and WNT5A showed relatively broad expression patterns across multiple clusters, whereas certain androgen-responsive genes exhibited more restricted expression (Supplementary Figure S1K).

Together, these findings illustrate the complex transcriptional landscape of the developing genital tubercle and highlight coordinated activity of developmental and androgen-related signaling pathways during formation of the male external genitalia.

### 3.3. Contextual Reference to Steroidogenic Pathways in a Public Mouse Leydig Cell Dataset

To investigate transcriptional programs associated with Leydig cell differentiation, RNA sequencing data from the Leydig differentiation model (GSE275902) were analyzed. Gene expression counts were normalized to counts per million (CPM) and log-transformed prior to analysis.

Principal component analysis (PCA) was performed using a panel of genes associated with androgen signaling and steroidogenesis. This analysis revealed clear transcriptional separation between control and knockout samples along the first principal component, indicating substantial differences in the transcriptional programs associated with Leydig cell differentiation (Supplementary Figure S2A).

Expression patterns of steroidogenic genes were further examined using heatmap visualization. Several genes involved in androgen biosynthesis showed coordinated expression patterns across samples (Supplementary Figure S2B).

Statistical analysis using Welch’s *t*-test followed by Benjamini–Hochberg correction identified significant downregulation of multiple steroidogenic genes in knockout samples. In particular:

CYP11A1 (FDR = 1.1 × 10^−2^)

HSD3B1 (FDR = 2.4 × 10^−2^)

INSL3 (FDR = 6.0 × 10^−3^)

were significantly reduced compared with control samples.

Among these genes, INSL3 exhibited the largest reduction in expression. INSL3 is produced by differentiated Leydig cells and serves as a key marker of Leydig cell maturation (Supplementary Figure S2C). Visualization using boxplots confirmed the reduction in these genes in knockout samples. In contrast, STAR, an upstream regulator of steroidogenesis responsible for mitochondrial cholesterol transport, showed only a modest decrease in expression and did not reach statistical significance.

Taken together, these results indicate transcriptional disruption of the steroidogenic gene network associated with Leydig cell differentiation.

## 4. Discussion

Hypospadias is widely considered a developmental disorder of the external genitalia arising from incomplete fusion of the urethral folds during embryogenesis. The severity of the defect depends on the location of the urethral meatus, with perineal hypospadias representing the most severe form of the condition. In dogs, this phenotype is frequently associated with additional abnormalities such as penile hypoplasia, cryptorchidism, and malformations of the prepuce.

The present case illustrates an unusual clinical course in which a dog with severe perineal hypospadias survived to geriatric age under conservative management without surgical correction. Although recurrent urinary tract infections occurred during early life, the patient remained clinically stable during the final years before presentation. Long-term management consisting of daily hygiene of the perineal region, intermittent antimicrobial therapy when necessary, and supportive nutritional care allowed maintenance of acceptable quality of life despite severe congenital malformation [20,21].

An important feature of this case was the presence of bilateral cryptorchidism and subsequent development of testicular tumors, specifically a Leydig cell tumor and a seminoma. Cryptorchid testes are known to have a substantially increased risk of neoplastic transformation. Seminomas arise from germ cells, whereas Leydig cell tumors originate from steroidogenic interstitial cells responsible for androgen production. The coexistence of these tumors in a patient with congenital genital malformation raises the possibility that common developmental mechanisms may link these conditions.

Embryonic formation of the external genitalia is strongly influenced by androgen signaling and by transcriptional programs governing genital tubercle morphogenesis [22]. Our analysis of single-cell RNA-sequencing data from embryonic genital tubercle tissue revealed expression of several key developmental regulators, including HOXA13, HOXD13, SHH, WNT5A, FGF8, and BMP7. These genes are well known to coordinate patterning and morphogenesis of the external genitalia. Disruption of these pathways has been implicated in human hypospadias and related developmental disorders.

In addition to developmental signaling pathways, androgen production by Leydig cells is critical for normal male reproductive development [23]. Steroidogenic enzymes such as CYP11A1 and HSD3B1 catalyze essential steps in androgen biosynthesis, while the peptide hormone INSL3 produced by Leydig cells plays a crucial role in testicular descent. Reduced INSL3 signaling has been associated with cryptorchidism in several species [24,25].

Evidence from previously published transcriptomic studies of Leydig cell differentiation indicates that several steroidogenic genes, including CYP11A1, HSD3B1, and INSL3, may be downregulated under conditions of impaired Leydig cell maturation. Among these, INSL3 is recognized as a key regulator of testicular descent, and alterations in this pathway have been implicated in cryptorchidism, although this was not directly evaluated in the present case.

Recent human genomic studies have also implicated dysregulation of steroidogenic and androgen-related genes in hypospadias [26]. This is consistent with the present findings demonstrating coordinated downregulation of key steroidogenic genes, including CYP11A1, HSD3B1, and INSL3. Beyond steroidogenic pathways, recent sequencing-based studies have highlighted the involvement of disorders of sex development (DSD)-related genes in the pathogenesis of hypospadias. These findings suggest that hypospadias may arise from broader dysregulation of sex differentiation and gonadal development programs, rather than isolated alterations in androgen biosynthesis alone. In this context, the coexistence of hypospadias and cryptorchidism in the present case may reflect overlapping developmental disturbances rather than independent anomalies.

In the present case, the combination of severe perineal hypospadias, bilateral cryptorchidism, and subsequent development of testicular tumors underscores a clinically relevant association between congenital urogenital malformation and later-life reproductive pathology. Cryptorchid testes are known to be predisposed to neoplastic transformation, and the occurrence of both a Leydig cell tumor and a seminoma in this patient is consistent with previous observations in veterinary medicine.

Because no molecular data were obtained directly from the reported animal, the transcriptomic datasets referenced in this study were used only as general biological context. All transcriptomic data were derived from mouse models, and given known interspecies differences in genital development, Leydig cell biology, and reproductive pathology, direct extrapolation to canine biology should be approached with caution.

Accordingly, the findings of this study are best interpreted as descriptive and hypothesis-generating rather than as evidence of a direct mechanistic relationship. The transcriptomic component was included solely to provide a biologically informed framework for interpreting the observed clinical phenotype, rather than to establish novel molecular insights or species-specific conclusions.

From a clinical perspective, this case also demonstrates that conservative management may be feasible in selected patients with severe hypospadias. Despite the presence of a significant congenital anomaly, long-term survival with acceptable quality of life was achieved through careful hygiene of the perineal region, monitoring for urinary tract infections, and supportive care. Such management strategies may represent a reasonable alternative when surgical correction is not feasible or when the patient has adapted well to the congenital defect.

However, clinicians should remain aware that animals with congenital urogenital anomalies, particularly those with cryptorchidism, may remain at increased risk for reproductive tumors later in life [27]. Continued monitoring is therefore recommended in such patients.

## 5. Conclusions

This study describes an unusual presentation of severe perineal hypospadias with long-term survival under conservative management and subsequent development of bilateral testicular tumors in a dog. The findings highlight a clinically relevant association between congenital urogenital malformation and later-life reproductive pathology.

To support biological interpretation, publicly available transcriptomic datasets related to genital development and Leydig cell function were incorporated as contextual reference. These datasets illustrate established developmental and endocrine pathways relevant to urogenital development, but do not constitute direct molecular evidence from the reported animal.

Accordingly, this study should be regarded as a clinically grounded integrative analysis that combines detailed case observation with biologically informed interpretation. The conclusions are therefore descriptive and hypothesis-generating, rather than evidence of a direct mechanistic relationship between hypospadias, cryptorchidism, and testicular tumor development.

## Data Availability

Publicly available datasets were analyzed in this study. Single-cell RNA sequencing data of embryonic genital tubercle tissue are available in the Gene Expression Omnibus (GEO) under accession number GSE175498 (https://www.ncbi.nlm.nih.gov/geo/query/acc.cgi?acc=GSE175498, accessed on 11 March 2026), RNA sequencing data of the Leydig cell differentiation model are available in GEO under accession number GSE275902 (https://www.ncbi.nlm.nih.gov/geo/query/acc.cgi?acc=GSE275902, accessed on 11 March 2026). Additional processed data generated during the current study are available from the corresponding author upon reasonable request. Processed data and analysis scripts are available from the corresponding author upon reasonable request.

## Supplementary Materials

The following supporting information can be downloaded at: https://www.mdpi.com/article/10.3390/cimb48050455/s1, Figure S1: Single-cell transcriptomic landscape of genital tubercle development (GSE175498). Figure S2: Transcriptomic disruption of the Leydig steroidogenic axis.
