# Supplementary material for: Clinical Course of Severe Perineal Hypospadias with Cryptorchid Testicular Tumors in a Dog: Contextual Reference to Developmental and Endocrine Transcriptomic Pathways
Source: Curr Issues Mol Biol. 2026 Apr 28;48(5):455. doi: 10.3390/cimb48050455 (PMC13206638; doi:10.3390/cimb48050455)
Supplement: Supplementary file 1 [file cimb-48-00455-s001.zip › cimb-4281132-Supplementary.pdf]

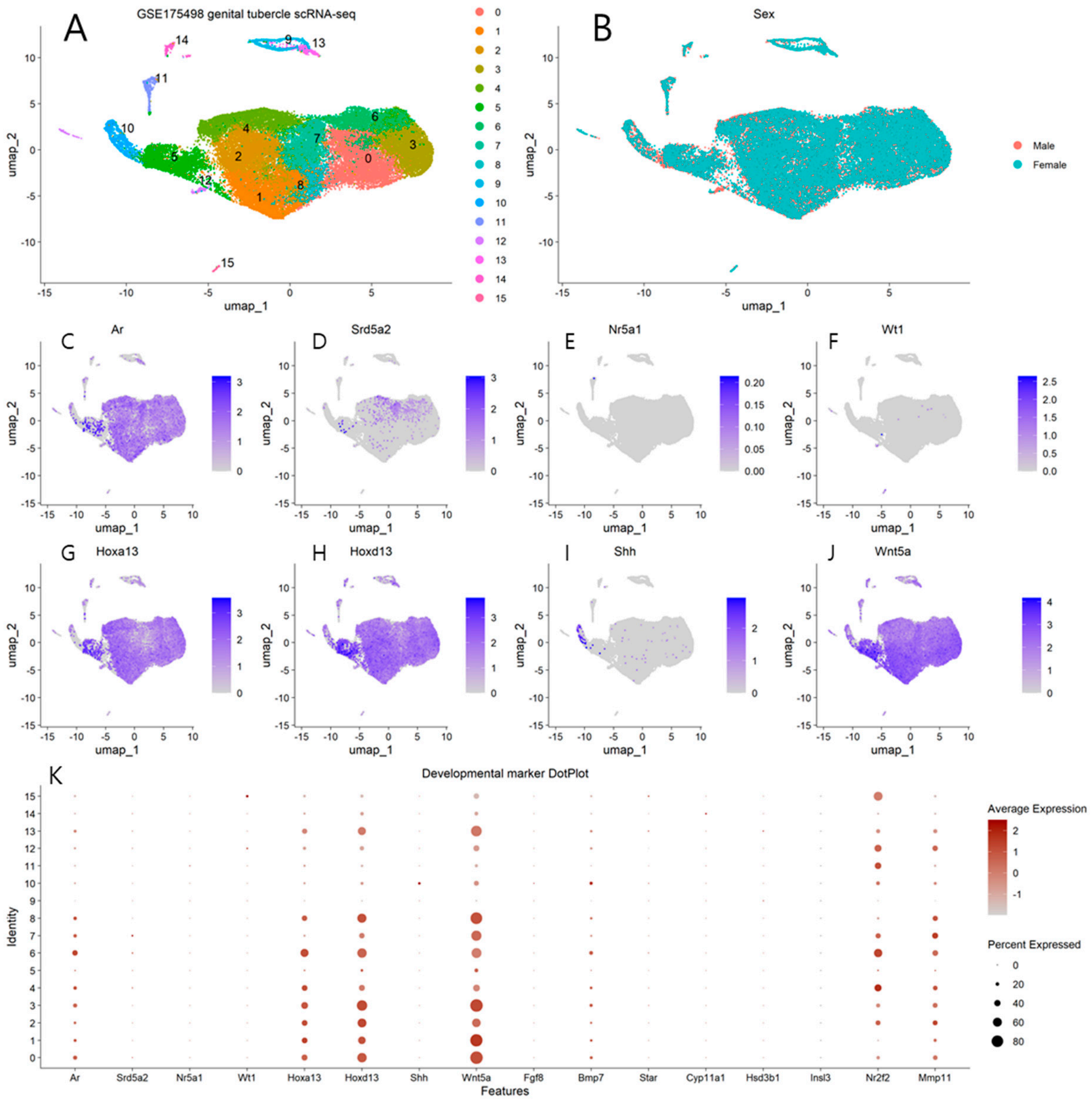

Figure S1. Single-cell transcriptomic landscape of genital tubercle development (GSE175498). This dataset is derived from a mouse model and is presented for contextual illustration of developmental pathways. (A) UMAP visualization of single-cell RNA sequencing data from embryonic genital tubercle tissue after quality-control filtering. A total of 43,827 cells were retained and grouped into 16 transcriptionally distinct clusters. (B) UMAP plot colored by sex demonstrating substantial overlap between male and female cells, indicating that early transcriptional programs of genital tubercle development are largely shared between sexes at this developmental stage. (C–J) FeaturePlot visualization of representative developmental and androgen-related genes. Developmental patterning genes including HOXA13, HOXD13, SHH, WNT5A, FGF8, and BMP7 showed distinct spatial expression patterns across clusters. Expression of androgen-related genes such as AR, SRD5A2, NR5A1, and WT1 was also detected in specific cellular populations. (K) Dot plot summarizing expression patterns of developmental and steroidogenic marker genes across

clusters. Dot size represents the percentage of cells expressing the gene, and color intensity represents the average expression level.

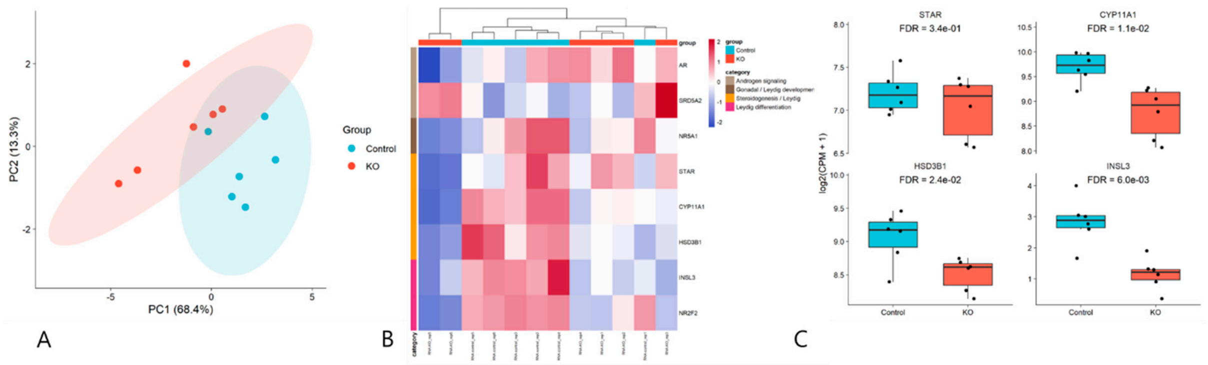

Figure S2. Transcriptomic disruption of the Leydig steroidogenic axis. This dataset is derived from a mouse model and is presented for contextual illustration of endocrine pathways. **(A)** Principal component analysis (PCA) based on androgen signaling and steroidogenic genes showing transcriptional separation between control and knockout (KO) samples. The first principal component explains 68.4% of the variance and distinguishes the two experimental conditions.**(B)** Heatmap showing expression patterns of genes associated with androgen signaling and Leydig cell differentiation, including AR, SRD5A2, NR5A1, STAR, CYP11A1, HSD3B1, INSL3, and NR2F2. Samples are grouped according to experimental condition (Control vs. KO), and color intensity represents scaled gene expression values.**(C)** Boxplots showing expression levels of representative steroidogenic genes STAR, CYP11A1, HSD3B1, and INSL3. Statistical significance was assessed using Welch's t-test with Benjamini–Hochberg false discovery rate (FDR) correction. Significant reductions were observed for CYP11A1 (FDR = 1.1×10^{-2}), HSD3B1 (FDR = 2.4×10^{-2}), and INSL3 (FDR = 6.0×10^{-3}) in KO samples.
